# Pharmacy Technicians Help to Push Boundaries in Delivering Quality Care

**DOI:** 10.3390/pharmacy8020098

**Published:** 2020-06-07

**Authors:** Shane P. Desselle, Kenneth C. Hohmeier

**Affiliations:** 1 California College of Pharmacy, Touro University, Vallejo, CA 94592, USA; 2 College of Pharmacy, University of Tennessee Health Sciences Center, Nashville, TN 37211, USA; khohmeie@uthsc.edu

We are so pleased that *Pharmacy* dedicated a themed Special Issue to pharmacy workforce supportpersonnel, namely technicians. Pharmacy technicians are increasingly recognized for their roles in supporting the delivery of pharmacy care services. The literature of pharmacy technicians has swelledin the past few years, with too many instances of their involvement to entirely enumerate here. We will point out a recently published systematic review that illuminates pharmacy technicians’ evolving role in medication therapy management (MTM) [1]. That review underscored the importance of pharmacy technicians in assisting with medication reconciliation and the documentation of services provided. Another study has proffered a new paradigm of practice, the Optimizing Care Model, which leveragesthe concept of tech-check-tech into an organizational culture and workflow redesign concept that helps maximize technicians’ effectiveness and allows pharmacists to expand their autonomy [2]. In fact, this study has served as the basis for practice change in one U.S. state to facilitate delegation to pharmacy technicians and has the support of the National Association of Chain Drug Stores, which might drive yet further change more rapidly [3].

It is anticipated that the articles published in this Special Issue will help further advance pharmacy care both in the U.S. and globally. Two of the articles in this themed issue demonstrate the effectivenessof integrating pharmacy technicians into medication synchronization (med sync) programs. Med sync programs have the potential to greatly improve medication adherence, and their success will be aided by further standardization of operational processes [4].Among those processes include a redefinition of technician roles and the use of appointment-based models (ABM) to facilitate patient interaction and prospective drug utilization review. The first of the two articles on med sync ABM employedqualitativeresearch to describe technicians’ job descriptions for this type of service [5]. The tasks in which techniciansmight assume greatest responsibility include identifying patients for marketing and enrollment, reviewing patients’ medication lists, choosing alignment dates based on patient preference, contacting patients in preparation for dispensing, and engaging in pickup or delivery of medications. A second study employed the Consolidated Framework for Implementation Research (CFIR) to examine not only initial design, but the sustainability of med sync ABM services [6]. The study found that among pharmacy technicians engaged in helping coordinate and deliver these services, in an absence of proper planning for workflow and job redesign, that other tasks might sometimes get short shrift; however, they expressed confidence that minor system flaws can be adjudicated and expressed considerable enthusiasm for their role in helping patients.

Another study in this issue took a holistic approach in examining the evolving roles of pharmacists, technicians and other support personnel, concurrently [7].The study authors demonstrated an increase in the amount of time spent by pharmacists in direct patient care through a segmentation analysis that also bore witness to these pharmacists better integrating those direct patient care activities with distributive functions as well as use of remote locations to provide services. They emphasized the need for training, continuing education, monitoring, regulations, and process designs to keep up with these changes, and hopefully even get ahead of the curve. Boughen and Fenn provided similar optimism and words of advice in moving forward for technician practice in the United Kingdom (U.K.) [8]. The authors described a model of technician education that employed pyramidal structures of technician activities, beginning with assembling medications, up to advanced communication, coupled with education standards that begin with basic customer service and lead up to assistance with medication optimization. They also call for additional research that aims to optimize evolving technician roles in the context of improving patient safety.

In regard to education, Denmark has always been a beacon for its training of their pharmaconomists, who study for three years and require an in-residence component in a national program. Among other rather advanced education paradigms, pharmaconomists receive extensive training in patient communication. El-Souri et al. document how the advanced communication skills of pharmaconomists result in their ability to have identify large numbers of potential and actual drug-related problems during patient consultation, along with the ability to actually resolve many of those problems themselves [9]. The authors suggest that future studies be geared toward affecting policies that might advance patient safety that much further. The training that pharmaconomists receive in Denmark is even further enhanced by strategies often being employed in other health professions programs. In one study noted here, pharmaconomists in training tackled research problems with the requirement that they perform and make formal presentations on their findings, thus sharpening their reasoning, literature review, and communication skills, all while using the opportunity to remain abreast of key pharmacy issues ongoing regionally and throughout the world [10].

The issue of technician education and training has indeed received considerable attention aroundthe world. In the U.S., more states are requiring certification, particularly in light of what has been regarded as uneven quality in technician vocational programs [11]. In this Special Issue, Desselle et al. ascribed value to a national certification process adjudicated by either of two vendors [12].Pharmacists witness first-hand the value of certification, but especially when it is combined with other education and better linked with formal on-the-job training programs. Pharmacists in the study recognize the contribution of certification for imparting greater professionalism and more advanced knowledge in basic pharmacology and math skills, but recognize the need for certification processes to include more “soft skills” such as advanced communication, leadership, and ethical decision-making. It is hoped that technicians being better prepared and deploying such skills will inspire greater confidence among pharmacists and delegating emerging responsibilities to them. The findings of Jetha et al. further corroborate this notion, suggesting that further integration of such skills into technician education and training is necessary to maximize the benefit of future technician deployment and regulation [13]. An official from one of the certifying organizations in the U.S. speaks to this, and also indicates that they are working with various stakeholders, such as large employers and regulators to improve technician mobility across various settings in considering these basic skills, but also within organizations, such as with career laddering mechanisms [14].Career ladders provide hope and aspirations to employees that they might make increasingly substantial contributions to the organization and fulfill personal self-actualization goals. The move toward career laddering for technicians has been called for in otherresearch [15,16]. It might very well be demonstration of advanced skills, such as communication, upon which employers decide to use as a basis for job mobility and career laddering, rather than more technical tasks.

Being on the proverbial “front line”, technicians are the persons most likely to come into contact with patients with questions around use of opioids, complementary medicine, and a growing array of products transitioning to over-the counter status [17]. Technicians must have the judgment and temperament to field those sensitive questions and know which ones to triage to pharmacists. These are all part of the move toward professional socialization and enhancement of more global competencies among technicians, andare concurrent with the expanding role of the pharmacist in patient care. In this themed issue, Draime et al. analyzed 14 days’ worth of job advertisement listings for pharmacy technicians [18].Among the more common sought-after job qualifications were communication, office etiquette, and professionalism. This comports with other recent studies demonstrating a desire by both technicians and their employers for enhanced professionalization and socialization into the field, so as to make it more endearing as a life-long career [19,20].

Policymakers and regulators must be more nimble and proactive in anticipating needed practice change. A study in Wales saw increases in technicians assuming leadership roles [21]. The study authors recommend not only that technicians seek development opportunities in this area but also that pharmacists become more adept at the art of delegation and work with profession leaders to optimize appropriate staffing levels and skills mix of support personnel to advance practice. Eid et al. pointed out that U.S. state board of pharmacy regulations were often overly prescriptive and favored the term “not expressly prohibited” in regard to many pharmacy technician roles [22]. This might be an improvement over them being previously being prohibited. However, some states like Idaho have taken a different tact, where instead of listing functions that a technician can do, it lists a very succinct list of those that they cannot perform, and instead simply leave it up to the pharmacist to determine the scope of technician practice activities under their supervision [23]. As regulations must keep pace with practice change, so must salary and other economic levers. Zgarrick et al. point out that while technicians are being asked to do more, their salary is not keeping up with these extra responsibilities and additional stressors on the job [24]. They analyzed data from the Bureau of Labor and Statistics to find that technicians have not enjoyed any wage premiums; in fact, those wage premiums (expectations for pay above the rate of inflation) have been flat or even negative in the past decade, and express concern that technicians can sometimes find better-paying jobs among unskilled labor positions. It will be especially important that improvements be made in earning potential and career mobility, as we have likely seen only the beginning in a wave of changes for pharmacy technician practice. Technicians have been involved in coordinating immunization activities, but now are gaining approval as immunizers and are thus side-by-side with pharmacists in helping them to take center stage in public health initiatives [25]. Properly trained technicians can help prevent pharmacists from being overwhelmed during certain seasonal events such as influenza upticks and inspire confidence among patients that pharmacies are an appropriate place to seek health solutions.

The articles in this themed SpecialIssue help to underscore the importance of pharmacy technicians. They also show how far we have come in better integrating technicians into the support of pharmacist-led, patient-centered services. At the same time, we still have much room for improvement, and those issues and gaps are identified. The profession of pharmacy will advance only as far as its constituent workforce personnel advances as well.
